# Pandoraea Species Bacteremia in a Neonate: A Case Report

**DOI:** 10.7759/cureus.117203

**Published:** 2026-09-30

**Authors:** Syed Ali Raza Nasir, Kinza Junaid, Laraib Malik, Ali Shabbir Hussain

**Affiliations:** 1 Microbiology, Aga Khan University Hospital, Karachi, PAK; 2 Pediatric Infectious Diseases, Aga Khan University Hospital, Karachi, PAK; 3 Pediatrics and Child Health, Aga Khan University Hospital, Karachi, PAK

**Keywords:** bacteremia, burkholderia, neonate, pakistan, pandoraea

## Abstract

*Pandoraea* species are rare, nonfermenting Gram-negative bacilli that are increasingly recognized as opportunistic pathogens, particularly in patients with cystic fibrosis. They are often misidentified as *Burkholderia* or *Ralstonia* species and pose significant diagnostic and therapeutic challenges due to their variable resistance patterns and the absence of standardized susceptibility breakpoints. We report a case of a seven-day-old term, low-birth-weight neonate who presented with respiratory distress and features of sepsis. Blood cultures grew a nonfermenting Gram-negative organism, initially identified as *Burkholderia cepacia*. Further testing using automated identification systems confirmed *Pandoraea* species. The organism demonstrated reduced susceptibility to multiple empiric antibiotics, including meropenem and ceftazidime. In the absence of established interpretive criteria, antimicrobial therapy was guided by phenotypic susceptibility patterns and literature review, and treatment was modified to trimethoprim-sulfamethoxazole and levofloxacin. The patient showed gradual clinical improvement with resolution of clinical and laboratory markers of infection and was eventually discharged. This case highlights the importance of accurate identification of uncommon pathogens, awareness of their unique resistance profiles, and close interdisciplinary collaboration to guide management in the absence of standardized treatment guidelines.

## Introduction

*Pandoraea *species are infrequently encountered, nonfermenting Gram-negative bacilli belonging to the family *Burkholderiaceae*, increasingly recognized as opportunistic pathogens with notable diagnostic and therapeutic challenges. In routine laboratory practice, they may be difficult to identify and can be misclassified due to phenotypic overlap with other nonfermenters such as *Burkholderia *and *Ralstonia *species [1]. Although initially described in environmental settings including soil, water, and various animal-derived sources, *Pandoraea *has increasingly been recognized as a clinically relevant opportunistic pathogen [2]. Most reported human infections occur in patients with cystic fibrosis, particularly involving the respiratory tract [3]. Invasive infections caused by *Pandoraea *species remain rare but clinically significant, with manifestations including bacteremia, pneumonia, and, less commonly, deep-seated infections [3].

Neonatal bloodstream infection due to *Pandoraea *species is exceptionally uncommon and poses considerable diagnostic and therapeutic challenges. Management is further complicated by the absence of established interpretive susceptibility breakpoints and the organism’s variable resistance profile, which often includes reduced susceptibility to multiple classes of antibiotics. Given the rarity of reported *Pandoraea *species infections worldwide and the apparent absence of previously reported cases from Pakistan, this case highlights an unusual cause of neonatal sepsis and underscores the importance of accurate microbiological identification and susceptibility-guided antimicrobial therapy.

## Case presentation

A week-old female neonate was referred to our tertiary care neonatal intensive care unit (NICU) with respiratory distress (Day 1). She was a term baby delivered via elective lower-segment cesarean section due to fetal growth restriction, weighing 1.75 kg. The pregnancy was booked, and antenatal screening was unremarkable. Shortly after birth, she developed tachypnea and grunting, requiring continuous positive airway pressure (CPAP) support at the referring hospital. Empiric antimicrobial therapy with meropenem, colistin, and amikacin had been initiated prior to transfer. Upon admission, the baby remained tachypneic but hemodynamically stable. A full septic workup was performed, including blood cultures and lumbar puncture. Chest X-ray showed bilateral perihilar streaky shadowing with diffuse ground-glass haze. Respiratory support was continued with noninvasive ventilation, and empiric intravenous meropenem was maintained. The patient did not have any central venous lines on arrival. Initial blood cultures from the referring facility were negative.

Blood cultures sent on Day 1 flagged positive on Day 2, showing Gram-negative rods on Gram stain. Suspecting multidrug-resistant Gram-negative sepsis, intravenous colistin was added empirically. After 24 hours of inoculation of culture plates, the colonies on chocolate agar were small, round, and late oxidase-positive, and grew on MacConkey agar as non-lactose-fermenters. They were motile and did not ferment glucose. On disc diffusion testing, they showed resistance to aminoglycosides and polymyxin B. The organism was identified as *Burkholderia cepacia* complex by Day 4; however, there was a possibility of misidentification, as limited biochemical tests were performed. The baby continued to have fever spikes of 38°C despite being on meropenem, and serial laboratory investigations showed progressive leukocytosis, with a peak white blood cell count of 40 × 10⁹/L, and severe thrombocytopenia, with a platelet count of 23 × 10⁹/L, requiring platelet transfusion; hence, a "triple tap" (urine, CSF, and blood) was performed. Cerebrospinal fluid and urine cultures showed no growth.

Subsequent blood cultures sent on Day 3 and Day 5 showed growth of a similar organism as in the first blood culture (Figure 1). This time, microbial identification was performed using the automated VITEK 2 system (bioMérieux, France) with a VITEK 2 GN (Gram-negative) test card, along with an API 20NE (bioMérieux, France). The API 20NE showed positive reactions for assimilation of potassium gluconate, malate, trisodium citrate, and phenylacetic acid but did not provide a definitive bacterial identification, while the VITEK GN identification card identified the isolates as *Pandoraea *species in both cultures (excellent identification, 99% confidence). The VITEK 2 GN card showed positive reactions for L-proline arylamidase (ProA), L-lactate alkalinization (ILATk), glutamyl arylamidase pNA (AGLTp), succinate alkalinization (SUCT), tyrosine arylamidase (TyrA), citrate utilization (CIT), Ellman’s reaction (ELLM), and γ-glutamyl transferase (GGT).

**Figure 1:**
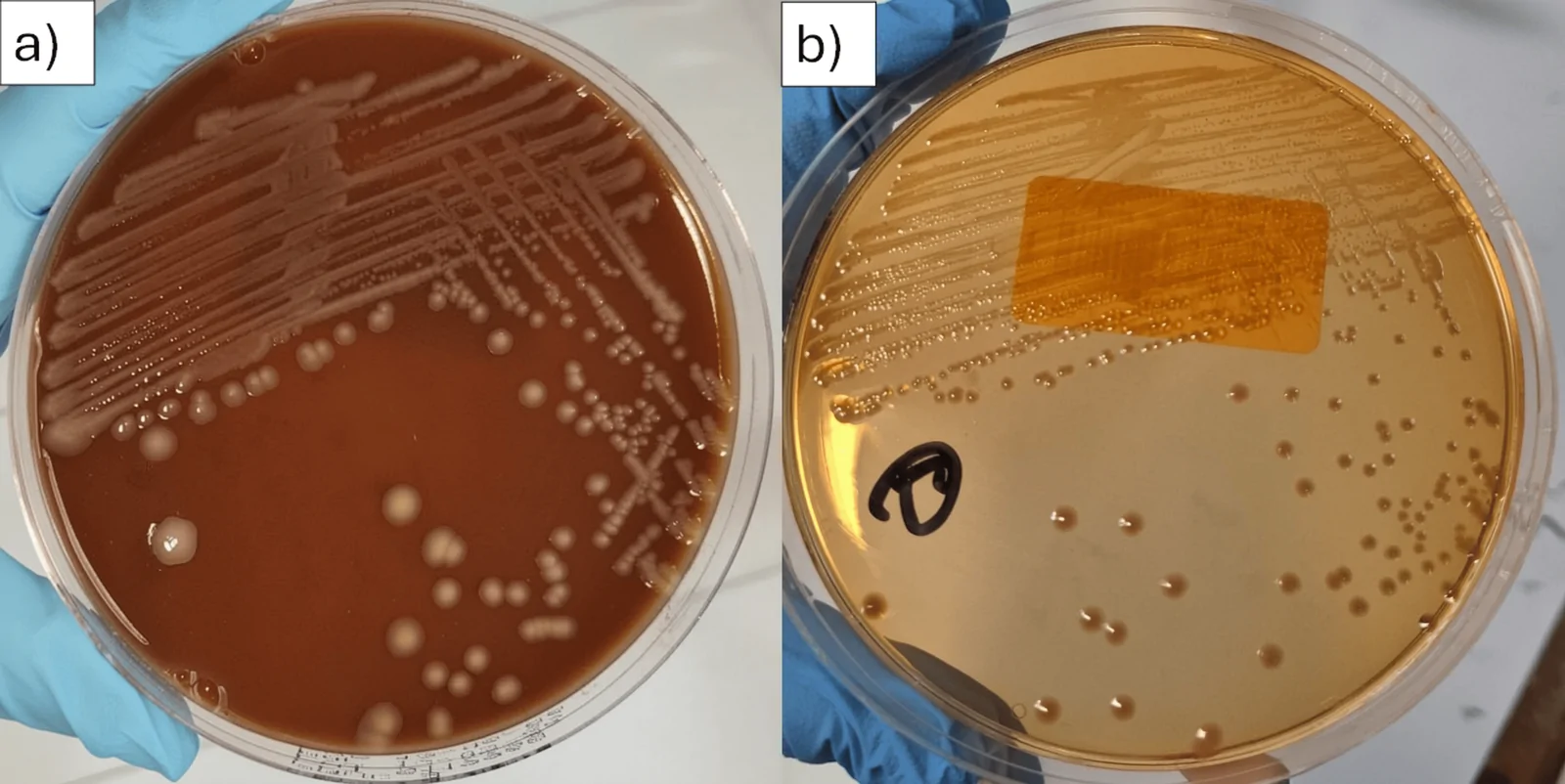
Colony morphology of Pandoraea species on (a) chocolate agar and (b) MacConkey agar Colonies on chocolate agar at 24 hours were small (1–2 mm), round, grey, convex, and had entire margins.

Disc diffusion testing and VITEK 2 susceptibility (AST-N419) results are shown in Table 1. Since there were no clinical breakpoints available, no susceptibilities were reported, and guidance on therapy was provided as comments in the report based on a literature review [3]. Subsequently, meropenem and colistin were discontinued, and targeted therapy was initiated with trimethoprim-sulfamethoxazole (Day 6) and levofloxacin (Day 7).

**Table 1:** Antimicrobial susceptibility testing of Pandoraea species CLSI clinical breakpoints are not available for *Pandoraea *species; therefore, categorical interpretations (susceptible, intermediate, or resistant) were not assigned. Zone diameters and MIC values are presented as observed. NP: Not performed; CLSI: Clinical and Laboratory Standards Institute; MIC: Minimum inhibitory concentration.

Antibiotic	Disc diffusion zone diameter (mm)	Minimum inhibitory concentration by VITEK 2 (µg/mL)
Amikacin	0	>64
Gentamicin	0	>16
Ciprofloxacin	25	>4
Meropenem	0	>16
Imipenem	26	2
Piperacillin/tazobactam	32	>128
Ceftazidime	12	>64
Trimethoprim/sulfamethoxazole	38	<20
Polymyxin B	0	NP

The baby showed gradual clinical improvement. Fever resolved, the respiratory distress subsided, and inflammatory markers showed a declining trend. Chest X-ray showed well-aerated lungs with no consolidations. Full enteral feeds were achieved, and respiratory support was successfully weaned. A peripherally inserted central catheter (PICC line) that was placed on Day 10 to administer antibiotics was removed by Day 14, and the baby remained clinically stable on room air. She was discharged home in a stable condition on Day 14 with oral levofloxacin and trimethoprim-sulfamethoxazole for 14 days post negative blood culture (sent on Day 7), with close outpatient follow-up arranged (Figure 2). The definitive source for this organism could not be established since no respiratory cultures were sent at any time during the admission, but a lower respiratory tract infection could possibly have been a source, considering the initial chest X-ray, the absence of a central venous catheter before the episode, and no isolation of *Pandoraea *in urine. She was planned to undergo a thorough investigation for cystic fibrosis on follow-up visits, which unfortunately could not be undertaken due to financial constraints of the family.

**Figure 2:**
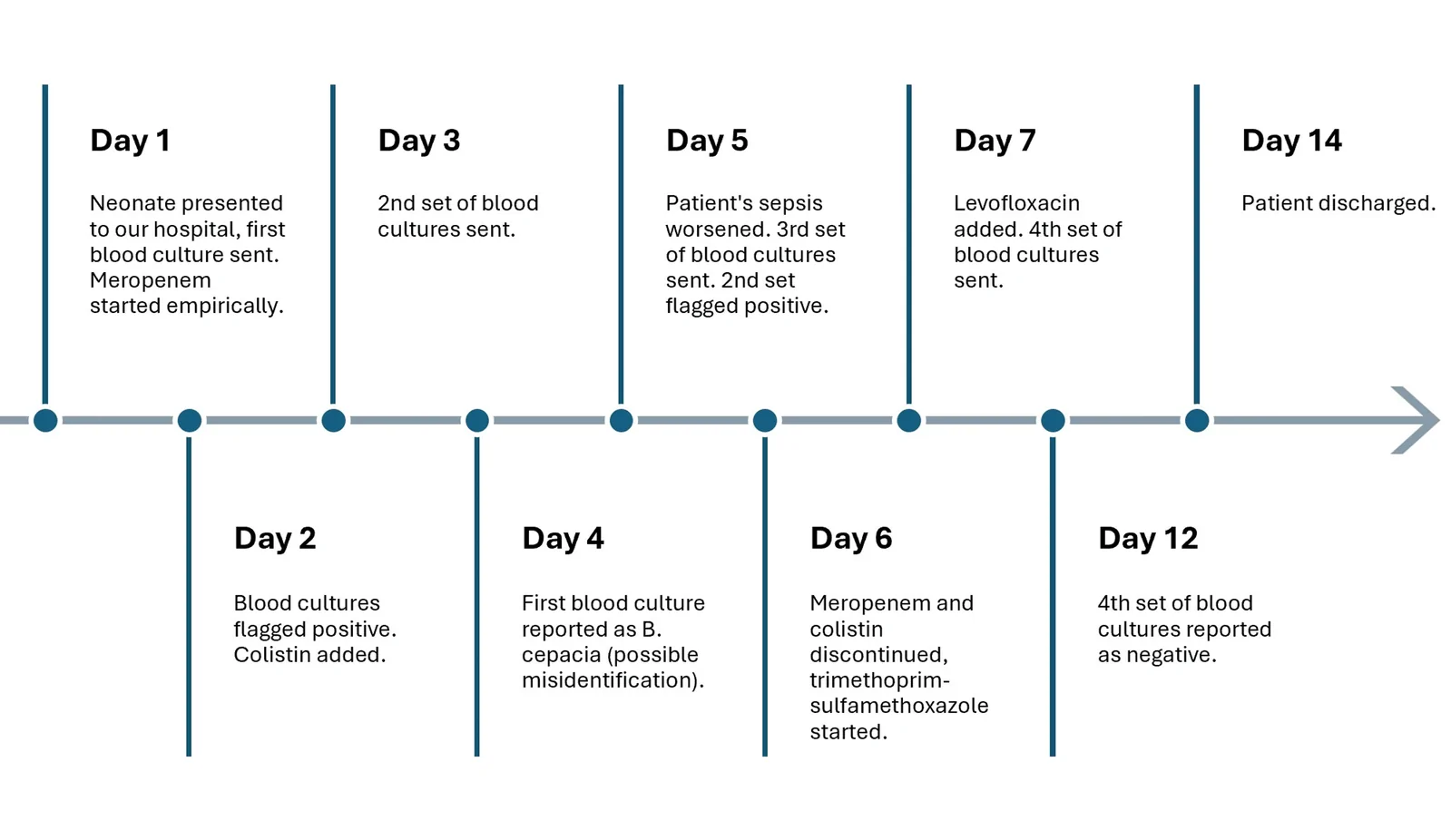
Timeline of events

## Discussion

*Pandoraea *species is an uncommon pathogen with an increasing number of infections being reported [3]. Our case represents one of the rare reported cases of *Pandoraea *infection from Pakistan and, to the best of our knowledge, one of the few reported cases in a neonate. Despite its rarity in the literature, it is reasonable to assume that true *Pandoraea *infections may be more common than appears, since the organism is frequently misidentified as *Burkholderia *and *Ralstonia*, as happened initially in our case [1]. Identification of *Pandoraea *spp. remains challenging because many commercial biochemical identification systems, including commonly used API databases, do not reliably identify members of this genus. Conventional biochemical tests are often insufficient to differentiate *Pandoraea *from other non-fermenting Gram-negative bacilli, leading to potential misidentification. Advanced identification methods such as matrix-assisted laser desorption/ionization time-of-flight mass spectrometry (MALDI-TOF MS), VITEK ID systems, or molecular techniques including 16S rRNA gene sequencing are generally required for accurate species-level identification. While our lab does have such resources, these technologies are expensive and inaccessible in many resource-limited laboratories across Pakistan.

Not only is the correct identification important for epidemiological reasons, but *Pandoraea *also has a distinctive susceptibility pattern. Notably, it is susceptible to imipenem but resistant to meropenem and is largely susceptible to trimethoprim-sulfamethoxazole (8.8% resistance) [3-5]. While CLSI (Clinical and Laboratory Standards Institute) or EUCAST (European Committee on Antimicrobial Susceptibility Testing) do not publish clinical breakpoints for *Pandoraea*, studies have reported high MICs of meropenem, cephalosporins, and quinolones [3]. This contrasts with *B. cepacia *complex, which remains largely susceptible to meropenem (around 90%) and ceftazidime (around 91%), and considerably resistant to trimethoprim-sulfamethoxazole (around 50%) [6,7]. However, there are several limitations to directly compare these figures, such as geographical differences in susceptibilities and the frequent changes in breakpoints for *B. cepacia *complex, while none are published for *Pandoraea *species.

The absence of established clinical breakpoints and limited access to reference methods such as broth microdilution complicate antimicrobial selection. In this case, antimicrobial susceptibility testing by disc diffusion demonstrated no zone of inhibition for meropenem, reduced susceptibility to ceftazidime, and comparatively larger zones for trimethoprim-sulfamethoxazole and ciprofloxacin. These findings, although lacking standardized interpretive criteria, were communicated to the clinical team and guided the initiation of trimethoprim-sulfamethoxazole in combination with levofloxacin. While this approach remains empiric and constrained by methodological limitations, it underscores the need for further studies incorporating genomic characterization to elucidate resistance mechanisms, correlating them with clinical outcomes and eventually establish clinically relevant breakpoints.

Recent studies into the virulence factors possessed by *Pandoraea *have revealed siderophore activity that offers iron scavenging to the bacteria, pandorabactins that have antibacterial activity against competing organisms, especially those colonizing the respiratory tract, and biofilm formation enabling it to colonize prosthetic material such as valves and central venous catheters [8,9]. This underscores the clinical importance of identifying *Pandoraea *to evaluate the need for source control, select antibiotics, and closely monitor patients.

The VITEK 2 system provides rapid identification; however, misidentification may occur with uncommon or closely related organisms due to limitations in its database. Therefore, results should be interpreted alongside clinical and conventional microbiological findings. In this case, the consistent biochemical profile obtained for two isolates from the same patient supports the reliability of the identification.

Overall, this case highlights the importance of considering *Pandoraea *as a potential cause of invasive infection, particularly when unusual susceptibility patterns or difficulties in organism identification are encountered. It also emphasizes the need for close collaboration among microbiology laboratories, infectious diseases specialists, and primary clinical teams to facilitate accurate diagnosis, guide antimicrobial therapy, and optimize patient outcomes.

## Conclusions

This case represents one of the rare reported neonatal bloodstream infections caused by *Pandoraea *species and one of the few cases described from Pakistan. It illustrates the diagnostic challenges posed by this uncommon pathogen, particularly its frequent misidentification as *B. cepacia* complex and the lack of standardized antimicrobial susceptibility breakpoints. Accurate laboratory identification using advanced diagnostic platforms was essential for recognizing the organism and informing antimicrobial selection in the absence of established treatment guidelines. The favorable clinical outcome following modification of therapy based on phenotypic susceptibility patterns highlights the value of close collaboration among microbiologists, infectious diseases specialists, and treating clinicians. Increased awareness of *Pandoraea *as a potential cause of invasive infection, together with further studies defining its antimicrobial susceptibility patterns and resistance mechanisms, will be important to improve diagnosis, guide therapy, and support the development of evidence-based management recommendations for this emerging pathogen.
